# Prevalence of *Brucella* endocarditis: A systematic review and meta‐analysis

**DOI:** 10.1002/hsr2.1301

**Published:** 2023-05-25

**Authors:** Negar Narimisa, Shabnam Razavi, Amin Khoshbayan, Faramarz Masjedian Jazi

**Affiliations:** ^1^ Microbial Biotechnology Research Center Iran University of Medical Science Tehran Iran; ^2^ Department of Microbiology, School of Medicine Iran University of Medical Sciences Tehran Iran

**Keywords:** *Brucella*, CMA, endocarditis, meta‐analysis, prevalence

## Abstract

**Background:**

Endocarditis caused by *Brucella* infection is one of this infection's complications, including a high mortality rate. However, studies on the prevalence of this complication have been limited to some case reports. This study investigated the prevalence of *Brucella* endocarditis globally using a systematic review and meta‐analysis.

**Methods:**

PubMed, Scopus, and Web of Science databases were searched using appropriate keywords until September 2022. All studies reporting the prevalence of endocarditis in patients with brucellosis were included in this current study. To investigate the pooled prevalence of *Brucella* endocarditis, random model was used in comprehensive meta‐analysis software.

**Results:**

A total of 25 studies met the inclusion criteria and were included in the systematic review and meta‐analysis. The prevalence of *Brucella* endocarditis was 1.3%, and the death rate was 26.5%. The results did not show a significant difference in the prevalence of this complication in different regions.

**Conclusion:**

According to this study's results, the prevalence of *Brucella* endocarditis is low, but it includes a large percentage of the deaths of affected patients. To complete our understanding of this complication and its management, more research should be done to investigate the effect of other factors, such as age and gender.

## 1 INTRODUCTION

Brucellosis is one of the most prevalent zoonotic diseases in the world. It is transmitted to humans directly through contact with bodily fluids from infected animals (sheep, goats, cattle, and other animals) or indirectly through derivative foods such as unpasteurized milk and cheese. Brucellosis causes high morbidity in both humans and animals.^1^, ^2^ It is a significant cause of economic loss and public health problems, especially in the Mediterranean and the Middle East.

Despite its low mortality rate, brucellosis remains a significant public health concern due to its long duration of treatment, slow recovery, and possible complications.^3^, ^4^

Brucellosis is a systemic disease that can affect any organ or system in the body. Clinical manifestations range from asymptomatic infection to a complete clinical picture of fever, night sweats, and joint pain. Complications vary greatly depending on the site of infection. Osteoarticular, reproductive, gastrointestinal, nervous, cardiovascular, skin and mucosa, and respiratory complications have been observed.^5^, ^6^

*Brucella* endocarditis (BE) is one of the most challenging complications of brucellosis, which is usually diagnosed late in the course of the disease with more involvement of the aortic valve and can affect both natural and artificial heart valves. Endocarditis due to brucellosis is rare, but most deaths from brucellosis are due to endocarditis. Moreover, it is one of the diseases that require rapid diagnosis and continuous evaluation of the treatment plan to evaluate whether the patient needs to repair the infected valve with surgery or whether drug treatment alone is adequate.^5^, ^7^

Because little information is available about the prevalence of BE. This study aims to investigate the prevalence of endocarditis in patients with *Brucella* infection.

## 2 MATERIALS AND METHODS

### 2.1 Search strategy

Three databases (PubMed, Scopus, and Web of Science) were searched until September 1, 2022. All these databases were searched by this search strategy: (Brucel*) AND (Endocarditis).

### 2.2 Inclusion criteria and exclusion criteria

All studies reporting the prevalence of endocarditis in patients with brucellosis were included in this current study.

All identified articles were pooled using the Endnote X20 Citation Manager software, and duplicate articles were removed before being reviewed. The citations were then uploaded to Rayyan, a citation sorting application.^8^ Animal studies and studies involving coinfection with other pathogens were excluded. In addition, review articles, case‐report studies, conference papers, book chapters, and articles written in languages other than English were excluded.

### 2.3 Study selection

Two independent authors reviewed titles and abstracts, and irrelevant articles were excluded. Full texts of potentially relevant papers have been obtained and were independently reviewed by two authors. All disagreements were discussed to reach a consensus.

### 2.4 Data curation

**Figure 1:**
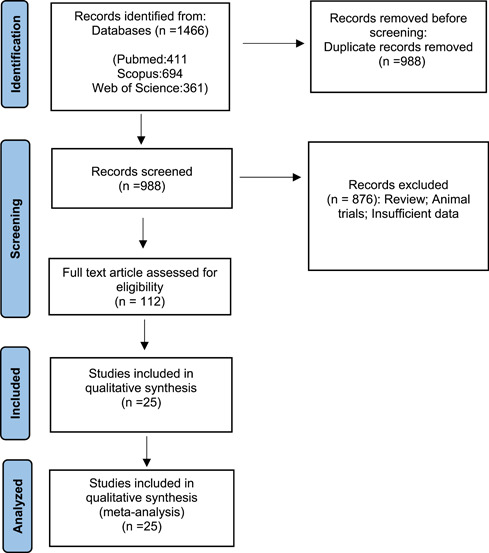
The study Prisma flow diagram.

For data extraction, two authors independently reviewed all eligible articles and extracted the following data: first author, publication year, study country, continent, and sample size (infected patients with brucellosis, those with endocarditis). A consensus and discussion resolved any disagreement between the researchers. This study selection process was presented in a Preferred Reporting Item for Systematic Reviews and Meta‐Analyses (PRISMA) flowchart (Figure 1).

### 2.5 Risk of bias assessment

The quality of included studies was evaluated using the Joanna Briggs Institute Critical Appraisal Checklist.

This prevalence survey assessment checklist includes nine basic questions. These questions focus on a suitable sampling frame, research objectives, and sufficient data analysis. Each item is rated yes, no, or unclear. A “yes” response was scored 1 point, and “no” and “unclear” responses were scored 0 points. Finally, the average score for each article was independently assessed by two reviewers, and disagreements were resolved by consensus.^9^

### 2.6 Data synthesis

Data analysis of the prevalence of endocarditis in patients with brucellosis was calculated with comprehensive meta‐analysis software. A random‐effects model was used to estimate the pooled prevalence of endocarditis in patients with brucellosis with 95% CI.^10^

Heterogeneity between studies included in the meta‐analysis was assessed using the *I* ^2^ statistic. *I* ^2^ ≤ 25% indicates low homogeneity and, 25% <*I* ^2^ ≤ 75% moderate heterogeneity, and *I* ^2^ > 75% indicated high heterogeneity.^11^

Sensitivity analysis was performed to examine the effect of removing individual studies to examine on the effect of the study on the overall result.^12^

Graphical and statistical methods are used to check for publication bias. A well‐known graphical method is the examination of funnel plots. The purpose of this method is to produce a scatterplot with the effect sizes on the horizontal axis and a measure of the size of each study on the vertical axis.^13^ The Bagg test is a statistical technique for assessing publication bias that assesses whether there is a significant correlation between the ranks of the effect estimates and the ranks of their variances. Results were assumed to have publication bias with *p* < 0.05.^14^

### 2.7 Subgroup analysis

Subgroup analyzes were performed for publication year, country, continent, and mortality frequency.

## 3 RESULTS

### 3.1 Study search

A total of 1466 publications were identified, and after reviewing, 988 studies were excluded, and 112 articles were analyzed for full‐text validation. After a full‐text review, 25 published studies met the inclusion criteria and were used for the meta‐analysis. Figure 1 illustrates the article review and selection process according to the PRISMA statement. And, characteristics of included studies are shown in Table 1.

**Table 1:** characteristics of included studies.

Name	Year	Country	Continent	Sample size (brucellosis)	Sample size (endocarditis)	Quality score
Abdi‐Liae et al.^15^	2006	Iran	Asia	85	3	8
Ahmetagic et al.^16^	2015	Bosnia and Herzegovina	Europe	246	1	7
Al Dahouk et al.^17^	2005	Germany	Europe	31	2	9
Al Shareef et al.^18^	2022	Saudi Arabia	Asia	90	6	6
Artuk and Gul^19^	2019	Turkey	Asia	220	1	9
Bouza et al.^20^	2002	Spaiin	Europe	75	0	8
Gonen et al.^21^	2013	Turkey	Asia	201	5	8
Guler et al.^22^	2014	Turkey	Asia	370	1	7
Hadadi et al.^23^	2014	Iran	Asia	450	3	5
Jia et al.^24^	2017	China	Asia	590	10	8
Keyvanfar et al.^25^	2021	Iran	Asia	104	0	8
Kochar et al.^26^	2007	India	Asia	175	1	7
Kokoglu et al.^27^	2006	Turkey	Asia	138	2	6
Kose et al.^28^	2014	Turkey	Asia	72	2	8
Lulu et al.^29^	1987	Kuwait	Asia	400	2	8
Megged et al.^30^	2016	Israel	Asia	105	1	9
Memish et al.^31^	2000	Saudi Arabia	Asia	545	3	7
Mermut et al.^32^	2012	Turkey	Asia	231	1	9
Öcal Demir and Becalan^33^	2020	Turkey	Asia	174	1	8
Pericherla et al.^34^	2021	India	Asia	94	1	6
Roshan et al.^35^	2004	Iran	Asia	469	3	6
Sathyanarayanan et al.^36^	2011	India	Asia	68	1	7
Asadi et al.^37^	2017	Iran	Asia	149	1	8
Türker et al.^38^	2014	Turkey	Asia	523	13	8
Xu et al.^39^	2020	China	Asia	285	3	7

### 3.2 Meta‐analysis

#### 3.2.1 Prevalence of BE

**Figure 2:**
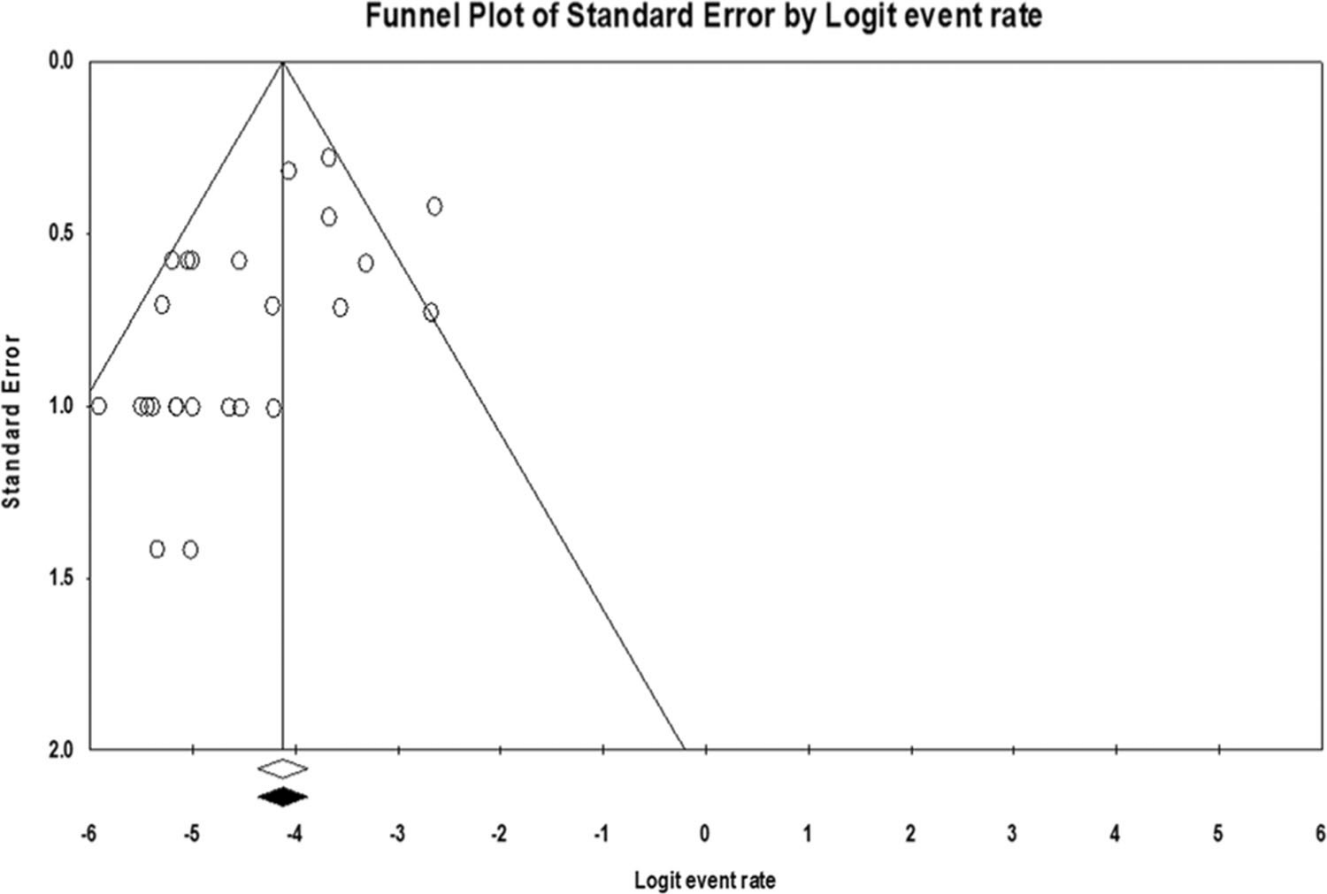
Funnel plot for identification of publication bias.

**Figure 3:**
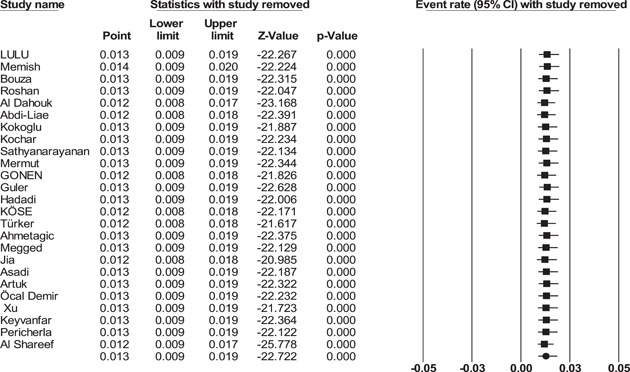
Forest plot for sensitivity analyses of prevalence of *Brucella* endocarditis.

Publication bias results were shown in funnel plots (Figure 2). In addition, Begg's test was used to indicate publication bias (*p* = 0.96). Sensitivity analysis was performed, and the result showed that none of the studies affected the prevalence of endocarditis, as shown in the forest plot of the sensitivity analysis (Figure 3).

**Figure 4:**
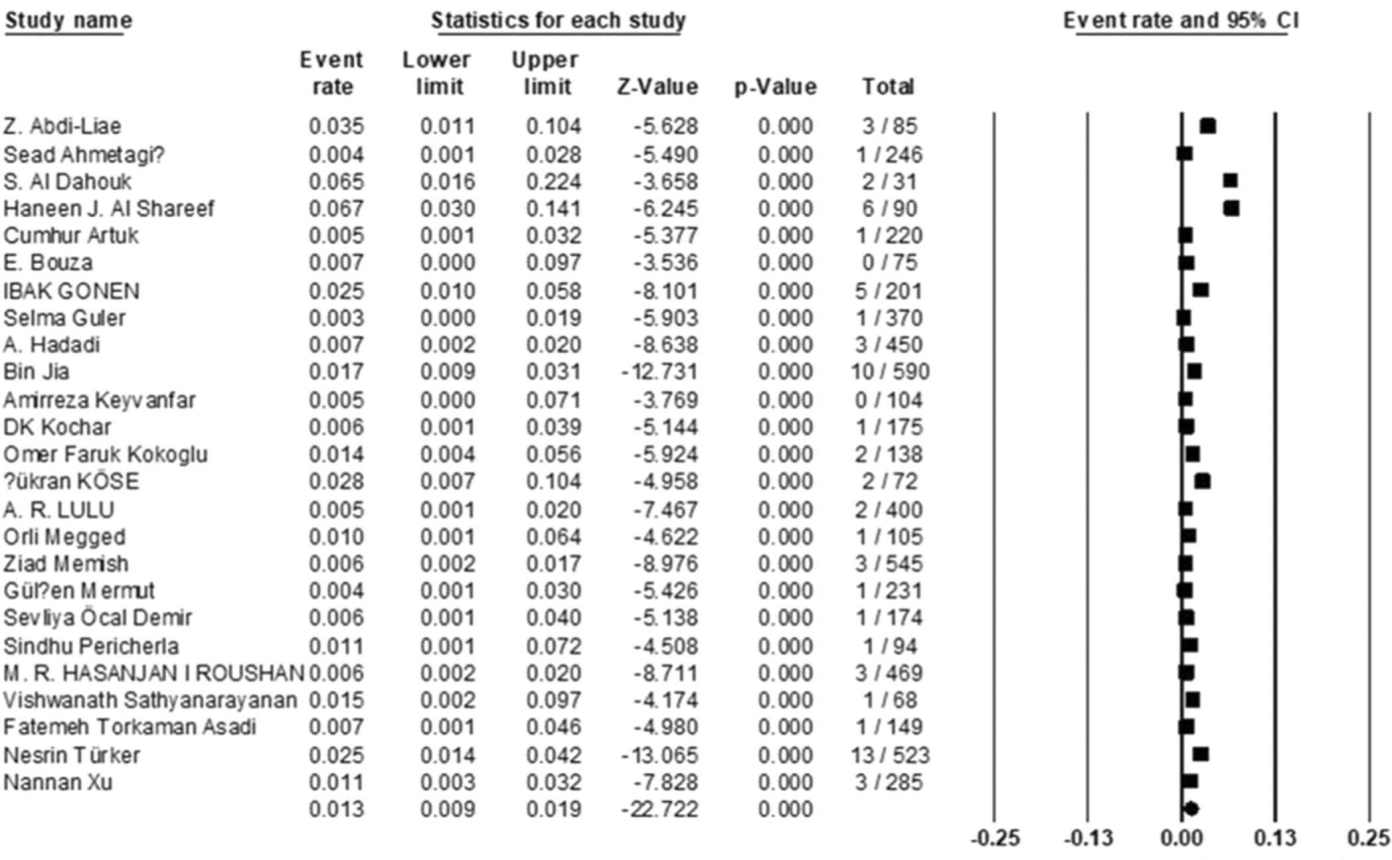
Forest plot showing the prevalence of *Brucella* endocarditis.

In this study, 5805 brucellosis cases from nine countries were investigated. The pooled prevalence of BE was estimated at 1.3% (95% CI: 0.9%−1.9%; *I* ^2^ = 48.8%; *p* = 0.003) (Figure 4).

### 3.3 Subgroup analysis

#### 3.3.1 Publication year of included studies

**Figure 5:**
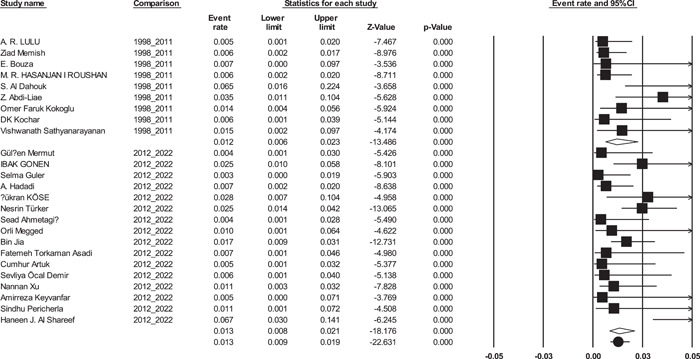
Forest plot showing the prevalence of *Brucella* endocarditis in two time period (1988−2011 and 2012−2022).

Subgroup meta‐analysis for publish year was performed in two periods (1988−2011 and 2012−2022). The year subgroup analysis indicated no significant changes of BE from 1988 to 2011 (1.2%; [95% CI: 0.06%−2.3%]) to 2012−2022 (1.3%; [95% CI: 0.08%−2.1%]), (*p* = 0.80) (Figure 5).

#### 3.3.2 Continent of included studies

**Figure 6:**
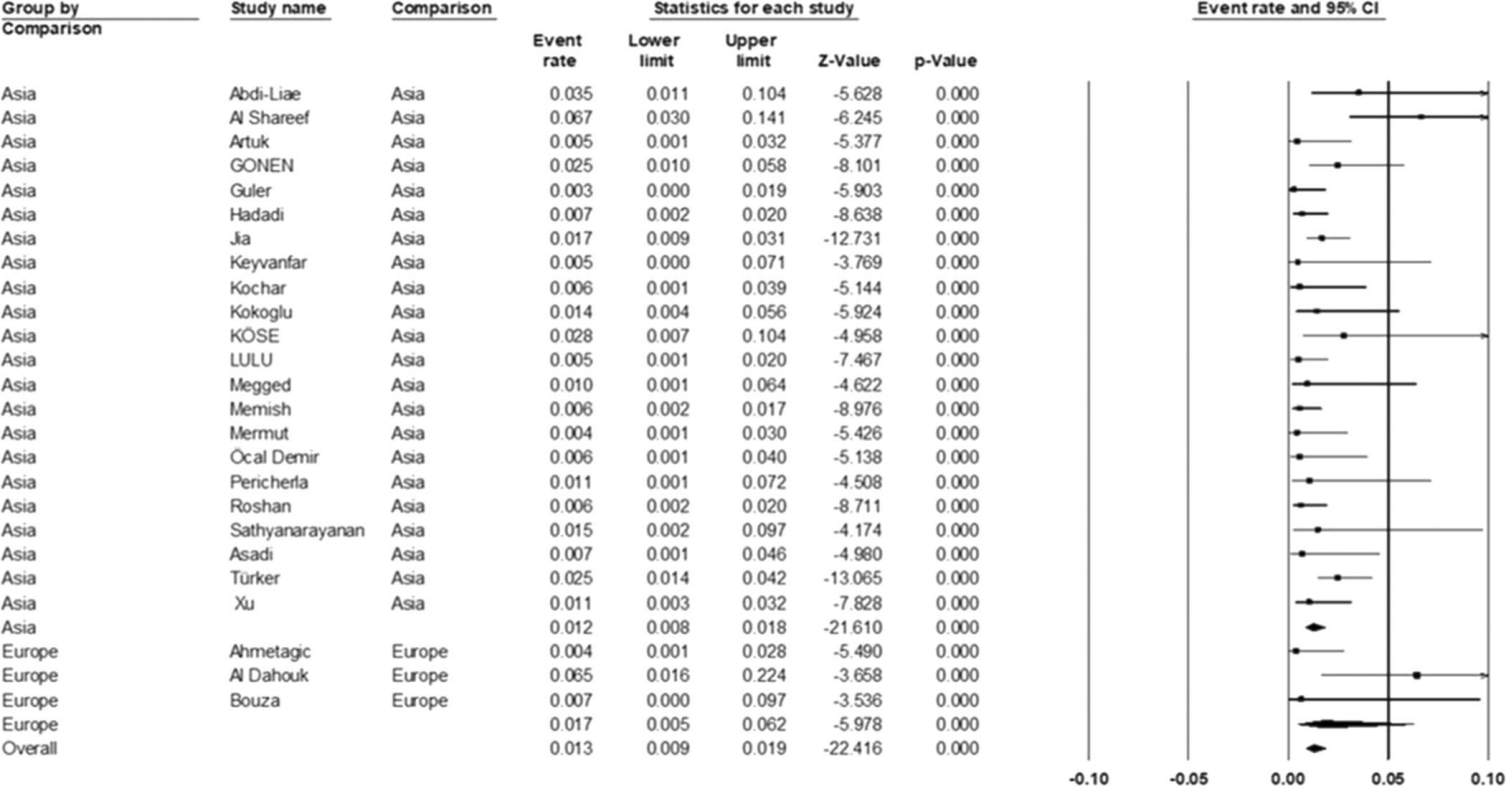
Forest plot showing the prevalence of *Brucella* endocarditis in different continents.

Among the 25 included studies, 22 studies were conducted in Asia and 3 studies were conducted in Europe, and the prevalence of BE was shown (1.2%; [95% CI: 0.8%−1.8%]) in Asia and (1.7%; [95% CI: 0.5%−6.2%]) in Europe (*p* = 0.62) (Figure 6).

#### 3.3.3 Country of included studies

**Figure 7:**
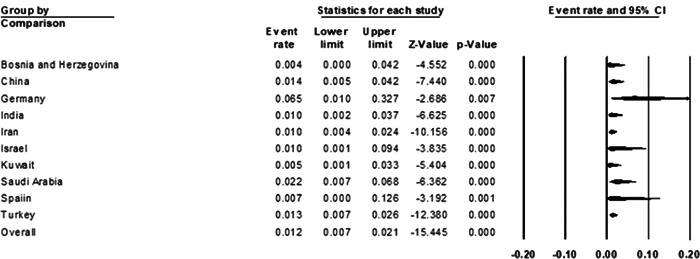
Forest plot showing the prevalence of *Brucella* endocarditis in different countries.

Of the 25 included studies, Bosnia and Herzegovina, Germany, Israel, Kuwait, and Spain each included 1 study, Saudi Arabia and China included 2 studies, India included 3 studies, Iran included 5 studies, and Turkey included 8 studies. Subgroup meta‐analysis of the country showed that Germany had the highest prevalence with (6.5%; [95% CI: 1%−32.7%]) and Bosnia and Herzegovina with (0.4%; [95% CI: 0.0%−4.2%]) had the lowest prevalence of BE, although only 1 study from these countries was included (Figure 7) (*p* = 0.70).

#### 3.3.4 Mortality frequency of BE

**Figure 8:**
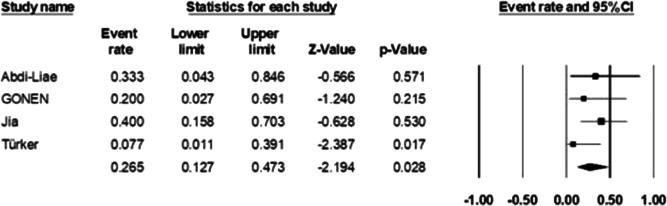
Forest plot showing the prevalence of death caused by *Brucella* endocarditis.

Four of the included studies had reported the number of deaths due to endocarditis in cases of Brucellosis, and the result of the analysis showed that the prevalence of death due to BE was 26.5% (95% CI: 12.7%−47.3%; *I* ^2^ = 2.13%; *p* = 0.38) (Figure 8).

## 4 DISCUSSION

This study aimed to systematically review the literature and perform a meta‐analysis to estimate the prevalence of BE. In this study, 25 articles that met the inclusion criteria were included in a systematic review and meta‐analysis.

Our results showed that the prevalence of BE is 1.3%. The largest included studies were from Turkey, Iran, and India. This result shows that although the prevalence of BE is low, it is still a significant health issue in these countries, emphasizing establishing preventive healthcare programs. Moreover, since eliminating brucellosis in humans is possible only by controlling the disease in animals, appropriate use of vaccines is needed to reduce the disease burden.

Although brucellosis is generally a problem in third world countries, especially countries in the Middle East, our results showed that the prevalence of endocarditis in the two continents of Asia and Europe is almost the same, with prevalence rates of 1.3% and 1.7%, respectively. These results emphasize that BE is a paramount public health concern in high‐risk and low‐risk areas.

We also checked whether the prevalence of BE has changed over the years. The analysis showed that the prevalence of this complication caused by brucellosis had almost the same pattern over the years, and we also checked the prevalence of endocarditis in two periods (1988−2011) and (2012−2022). Our results indicated that with a prevalence of 1.2 in (1988−2011) and 1.3 in (2012−2022), the prevalence of BE has not changed over time.

In our study, four articles reported the deaths of BE patients: In the study of Abdi‐Liae et al., 1 patient died out of 3 patients with BE. In another study by Gonen et al., 1 out of 5 patients with BE died. In the study of Jia et al., 10 patients with BE were identified. Six patients were treated with a good prognosis with antibiotics and valve replacement surgery. However, 4 other BE patients who did not undergo surgery died within 1 year of follow‐up. In the study by Türker et al., 1 patient died among 5 patients with BE who underwent mitral valve replacement. Although Peery et al.'s study in 1960 stated that the death rate of patients due to BE was 80%,^40^ the results of our analysis showed that the death rate of these patients was 26.5%.

There are limitations to this current study, for example, the lack of data to examine the prevalence of BE by age and gender. However, since there is no similar meta‐analysis study on the prevalence of BE, the current review is valuable and has helped to increase our understanding of the global prevalence of BE. Also, this study suggests that more research should be done to investigate the effect of age, gender, and other factors on BE to complete our understanding of this complication and its management.

## AUTHOR CONTRIBUTIONS

**Negar Narimisa**: Conceptualization; software; writing—original draft; writing—review and editing. **Shabnam Razavi**: Methodology; writing—review and editing. **Amin Khoshbayan**: Data curation; writing—review and editing. **Faramarz Masjedian Jazi**: Methodology; supervision; writing—review and editing.

## CONFLICT OF INTERESTS STATEMENT

The authors declare no conflict of interest.

## TRANSPARENCY STATEMENT

The lead author Faramarz Masjedian Jazi affirms that this manuscript is an honest, accurate, and transparent account of the study being reported; that no important aspects of the study have been omitted; and that any discrepancies from the study as planned (and, if relevant, registered) have been explained.

## Data Availability

All the data in this review are included in the manuscript.
